# PD24 Access To Mechanical Thrombectomy For Stroke In Uruguay Financed By National Resources Fund: Contribution Of Real-World Evidence

**DOI:** 10.1017/S0266462325101591

**Published:** 2025-12-29

**Authors:** Silvana Albisu, Abayuba Perna, Daniel Pedrosa, Eliana Lanzani

## Abstract

**Introduction:**

The National Resources Fund (FNR) is a financing agency for highly complex and high cost services. Among these is mechanical thrombectomy (MT) for acute ischemic stroke (AIS) due to large vessel occlusion. Quantifying access to this technique highlights the importance of continuing to identify and address barriers that limit the number of patients who can benefit from it.

**Methods:**

The study involved a retrospective cohort that included all the patients who applied for financing by the FNR from November 2021 to April 2024. It aimed to evaluate access to MT by select candidates for the procedure in accordance with current financial coverage regulations. To estimate the number of expected cases for Uruguay, previous epidemiological figures on the incidence of AIS (national studies) and data on hospital discharges with a diagnosis of AIS (provided by the Population Health Surveillance Area of the Ministry of Public Health of Uruguay for the year 2023) were considered.

**Results:**

In the period considered, 159 candidates for financing applied to FNR. Of these, 132 cases received full financing, five received partial financing, five didn’t meet the criteria for financial coverage, and 17 remained unresolved. Among the cases with full financing, 94 came from private health providers and 38 from public providers. Also, 81 cases originated in the capital’s geographical area and 51 in the interior of the country. Of the estimated annual number of people with AIS in Uruguay, 1.5 percent of them will have access to MT, for a base expected number of at least 3 percent according to real-world data.

**Conclusions:**

Access to MT financed by the FNR was lower than expected. To date, the number of patients that apply for financial coverage by the FNR remains stable at approximately 70 cases per year, with a significant disparity observed in geographical origin (Montevideo versus the interior) and in the patient’s healthcare provider of origin (public versus private).

